# Evaluating Compliance and Impact of the Sepsis Six Bundle in the Emergency Department: A Retrospective Cohort Study

**DOI:** 10.7759/cureus.96315

**Published:** 2025-11-07

**Authors:** Jixon Thomas, Parnika P Cherukupalli

**Affiliations:** 1 Emergency Medicine, Kottekad Catholic Dharmodaya Sangham Hospital, Thrissur, IND; 2 Internal Medicine, SRM Institutes for Medical Science (SIMS) Hospital, Chennai, IND

**Keywords:** emergency medicine, morbidity, mortality, sepsis, sepsis six bundle

## Abstract

Background and objectives

Sepsis resuscitation bundles are the subject of numerous quality improvement initiatives that have recently been implemented globally. These educational initiatives have been shown to be associated with improvements in clinical outcomes. The aim of this study was to evaluate the compliance and impact of the Sepsis Six bundle in the emergency department (ED).

Methodology

A cohort study was conducted among 660 patients who had been diagnosed with sepsis in the ED of a tertiary care center.

Results

The mean age of study participants was 44.8±11.7 years. Of the total, 37% of the patients were female and 63% were male. Among the study participants, 286 (43.3%) patients had the presence of shock; 220 (33.3%) had the presence of sepsis at the time of admission, and 440 (66.7%) had the presence of sepsis after 24 hours of admission. The primary site of infection in the majority of study participants was the lung, followed by the brain, haematological origin, and the kidney. A mortality rate of 32% was noted.

Conclusion

Targeted quality improvement measures, staff training, and streamlined ED workflows are warranted to enhance adherence to the Sepsis Six bundle and ultimately improve survival rates in septic patients.

## Introduction

When the body's immune system reacts excessively to an infection, it can lead to organ malfunction and sepsis, a potentially fatal illness. The body's reaction damages its own tissues and organs, and if it is not identified and treated quickly, it may result in shock, multiple organ failure, and even death. In 2020, sepsis accounted for 11 million deaths and 48.9 million cases globally, or 20% of all fatalities. Children under the age of five accounted for over half of all estimated cases of sepsis worldwide. An estimated 15 people out of every 1000 hospitalized patients will have sepsis as a side effect of their treatment [1].

Although sepsis can strike anyone in the world, there are notable regional differences in incidence and mortality, with lower-middle-income nations having the greatest rates [1]. In 2017, there were an estimated 11.3 million instances of sepsis in India alone, resulting in 2.9 million deaths (297.7 per 100,000 population) [2]. This emphasizes how important it is to step up efforts in LMICs like India to prevent, identify, and treat sepsis.

The Institute for Healthcare Improvement (IHI) created care bundles, which are condensed sets of evidence-based activities that, when carried out collectively, ought to produce better results than when carried out separately [3]. The Sepsis Six bundle, developed by the UK Sepsis Trust, is a set of six evidence-based interventions to be delivered within one hour of sepsis recognition. It is freely available for clinical and research use, provided appropriate attribution is given [4]. Limited information has been available regarding the sepsis six care bundle's compliance rates since its creation in 2007, and research on the bundle's effect on mortality rates has produced contradictory findings. When the bundle was first created in 2007-2008, an observational study demonstrated a mortality of 20% in patients receiving the Sepsis Six versus 44.1% in those who did not. [4]. There was no discernible difference in mortality associated with the administration of the entire Sepsis Six care bundle, despite the fact that only 12% of 290 sepsis patients in Wales received it, according to a recent study [5].

Both the National Confidential Enquiry into Patient Outcome and Death (NCEPOD) report [6] and the Parliamentary and Health Service Ombudsman report [7] suggest auditing compliance with sepsis care packages. Assessing adherence to the Sepsis Six care bundle is crucial in order to identify any obstacles to best practices and determine how this affects patient outcomes. Therefore, this study aimed to evaluate compliance with the Sepsis Six bundle and its association with patient outcomes in an Indian tertiary emergency setting. By following a group of patients diagnosed with sepsis over time and monitoring the implementation of the Sepsis Six bundle, the study was able to assess both adherence to protocol and its subsequent influence on measurable outcomes.

## Materials and methods

Study design

This study was designed as a cohort study aimed at evaluating the compliance with and clinical impact of the Sepsis Six bundle among patients presenting with sepsis in the Emergency Department (ED). A cohort design was considered appropriate because it allowed for systematic observation of patient characteristics, management, and outcomes within a real-world clinical setting, without interference from experimental allocation.

Study setting

The study was conducted in the ED of Kottekad Catholic Dharmodaya Sangham Hospital, Kottekad, Thrissur, Kerala, India, which serves as a referral center for a large catchment area. The ED is a high-volume unit that receives approximately 18,000 patients annually, including medical, surgical, and trauma cases. Given the busy nature of the unit and the frequency of sepsis cases encountered, it provided an ideal environment for examining compliance with time-sensitive interventions such as the Sepsis Six Bundle.

Study duration

The study was carried out over a defined period from October 2023 to April 2024. This duration ensured adequate patient recruitment, seasonal variation in infection prevalence, and sufficient follow-up to analyze short-term clinical outcomes such as antibiotic administration timeliness, hemodynamic stabilization, hospital length of stay, and mortality.

Study population

A total of 660 adult patients presenting with sepsis were included in the study. Patients were recruited consecutively upon diagnosis of sepsis, severe sepsis, or septic shock, according to the Sepsis-3 clinical criteria [8] and clinical judgment of the treating physician.

Inclusion Criteria

Adult patients aged 18 years or older presenting to the ED were included in the study. Eligible participants were those with a clinical diagnosis of sepsis, severe sepsis, or septic shock, defined by the presence of systemic inflammatory response syndrome (SIRS) criteria, along with suspected or proven infection and evidence of organ dysfunction attributable to sepsis. Only patients who met the criteria for initiation of the Sepsis Six bundle at the time of their arrival in the ED were included.

Exclusion Criteria

Patients transferred from other hospitals with ongoing sepsis management were excluded from the study, as prior treatment could confound the assessment of Sepsis Six bundle compliance at presentation. Those with incomplete or missing medical records that prevented accurate data collection were also excluded. In addition, patients who died or were discharged before the initiation of the Sepsis Six bundle were not included, as their early outcome precluded evaluation of compliance. Patients who declined or did not provide consent for participation in the study were similarly excluded. By applying these exclusion criteria, the study population was carefully defined to ensure homogeneity and reliability in the measurement of outcomes.

Sample size justification

The sample size of 660 patients was based on feasibility within the given study period and the availability of cases at the center. Given the relatively high incidence of sepsis in tertiary EDs, this number was sufficient to provide statistical power for evaluating both compliance rates and associations between bundle adherence and clinical outcomes. Additionally, the sample size allowed for stratified analysis of key variables such as age, sex, infection source, and presence of shock.

Data collection procedures

Data were collected prospectively from electronic health records, bedside charts, and direct observation where applicable. A structured data collection proforma was used by trained research assistants to ensure uniformity. Data points were collected at the time of admission to the ED and throughout the patient’s ED stay until transfer to inpatient wards or ICU.

The following information was recorded: (i) Demographics: Age, sex, race, and ethnicity, (ii) Clinical parameters at presentation: Day and time of arrival, designation of “time zero” (defined as the time of recognition of sepsis), day versus night shift categorization, initial vital signs, and presence of shock, (iii) Infection-related details: Suspected or confirmed source of infection (respiratory, urinary tract, intra-abdominal, skin/soft tissue, bloodstream, or other), (iv) Management variables: Time from recognition of sepsis to initiation of antibiotics, fluid resuscitation, blood cultures before antibiotics, lactate measurement, oxygen administration, and urine output monitoring (all components of the Sepsis Six bundle), (v) Outcome variables: Compliance with each individual element of the bundle, completion of the entire bundle within 1 hour, ICU admission, length of hospital stay, and in-hospital mortality.

Outcome measures

The primary outcome measure was the rate of compliance with the Sepsis Six bundle within one hour of time zero. Secondary outcomes included: (i) Timeliness of antibiotic administration (≤1 hour vs >1 hour), (ii) Completion of individual bundle elements (fluids, oxygen, blood cultures, lactate, antibiotics, urine monitoring), and (iii) Short-term outcomes such as requirement of ICU admission, length of hospital stay (measured in days), and in-hospital mortality.

Ethical considerations

Prior to commencement, the study protocol was submitted to and approved by the Institutional Ethical Committee of Kottekad Catholic Dharmodaya Sangham Hospital (reference number: KCDSH/TCR/IEC/512/2024-25). All participants or their legally acceptable representatives provided informed written consent before inclusion. Patient confidentiality was strictly maintained by anonymizing data during entry and analysis. The study adhered to the ethical principles outlined in the Declaration of Helsinki.

Data management and quality assurance

Data were initially entered into a Microsoft Excel spreadsheet (Microsoft Corporation, Redmond, Washington, United States) designed with in-built validation checks to minimize entry errors. The data were then cross-verified by an independent investigator to ensure accuracy and completeness. Missing or ambiguous entries were clarified from source documents wherever possible. Regular audits of data entry were conducted throughout the study period to maintain quality and consistency.

Statistical analysis

Statistical analysis was carried out using IBM SPSS Statistics for Windows, Version 26.0 (IBM Corp., Armonk, New York, United States).

Descriptive Statistics

Continuous variables were summarized as mean ± standard deviation (SD). Categorical variables were expressed as frequencies and percentages.

Inferential Statistics

The Chi-square test was used to compare categorical variables such as compliance rates across different demographic or clinical subgroups. One-way ANOVA was used for comparing means of continuous variables (e.g., time to antibiotic administration) between groups. Multiple logistic regression analysis was performed to identify independent predictors of bundle compliance and clinical outcomes such as mortality. A p-value < 0.05 was considered statistically significant. To ensure robustness, multicollinearity between variables was checked before inclusion in regression models, and adjusted odds ratios (OR) with 95% confidence intervals (CI) were calculated.

Rationale for Statistical Tests

The Chi-square test was chosen for categorical comparisons as it is appropriate for proportions, while ANOVA allowed comparison across more than two means. Logistic regression was included to control for potential confounders (e.g., age, sex, comorbidities) and determine the independent impact of Sepsis Six compliance on outcomes. This multi-level statistical approach enhanced the validity and reliability of the findings

## Results

A total of 660 patients diagnosed with sepsis and meeting the inclusion criteria were enrolled in the present study. The following observations and results were obtained from the analysis of their demographic characteristics, clinical parameters, infection source distribution, and outcomes.

Age distribution

The age distribution of the study population is presented in Table 1. The majority of patients were in the age group of 41-50 years, accounting for 220 (33.3%) participants. This was followed by 209 (31.6%) patients in the 31-40 years age range. A smaller proportion, 99 (15.0%), belonged to the 51-60 years category, while 44 (6.6%) patients were aged 21-30 years. The least represented group was patients aged >60 years, constituting 13.3% (n=88) of the study population.

**Table 1:** Distribution of study participants according to age group

Age group (in years)	Frequency	Percentages
21-30	44	6.6
31-40	209	31.6
41-50	220	33.3
51-60	99	15
> 60	88	13.3

Gender distribution

**Figure 1:**
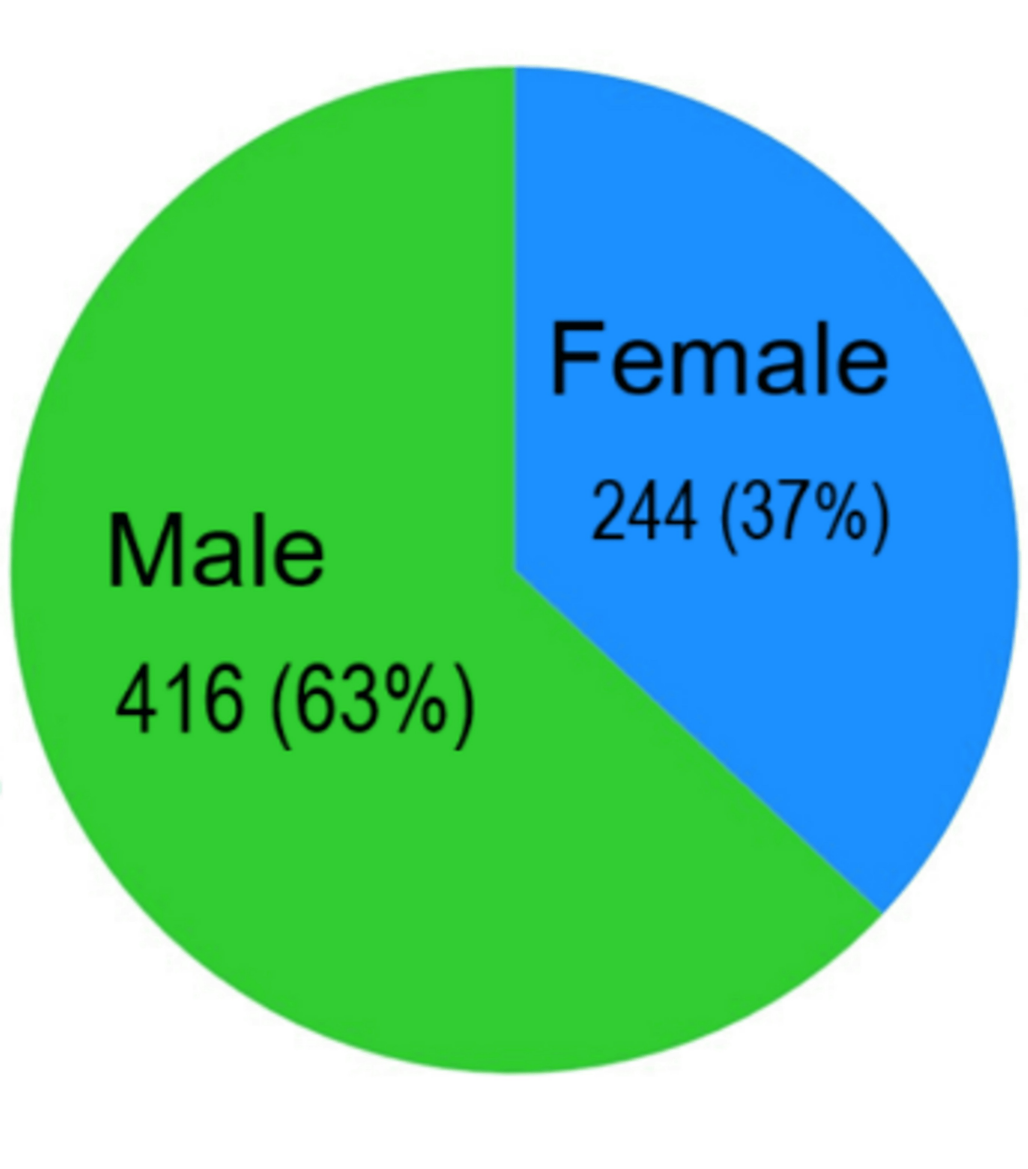
Gender distribution (N= 660)

Of the total 660 patients, 63% were male and 37% were female (Figure 1). This indicates a clear male predominance in the occurrence of sepsis among patients presenting to the ED. The observed gender distribution may reflect differences in healthcare-seeking behaviour, occupational exposures, comorbidities, or biological susceptibility to infection.

Presence of shock at presentation

Among the study participants, 286 patients (43.3%) presented with shock at the time of evaluation (Table 2). The presence of shock at admission is an important prognostic factor and indicates a severe state of sepsis requiring prompt and aggressive management. The relatively high proportion of patients with shock highlights the importance of early recognition and timely intervention, including implementation of the Sepsis Six bundle, in the ED setting.

**Table 2:** Distribution as per presence of Shock

Shock	Frequency	Percentages
Present	286	43.3
Absent	387	56.7

Timing of sepsis presentation

As shown in Table 3, of the total study participants, 220 (33.3%) had sepsis present at the time of admission to the ED. In contrast, 440 (66.7%) patients developed sepsis within 24 hours after admission. This finding suggests that a significant proportion of patients progress to sepsis during their early hospital course, underscoring the importance of vigilant monitoring and timely initiation of sepsis management protocols even in those who do not initially meet full sepsis criteria.

**Table 3:** Distribution as per time of sepsis presentation

Presence of Sepsis	Frequency	Percentages
At a time of admission	220	33.3
After 24 hours of admission	440	66.7

Primary site of infection

**Figure 2:**
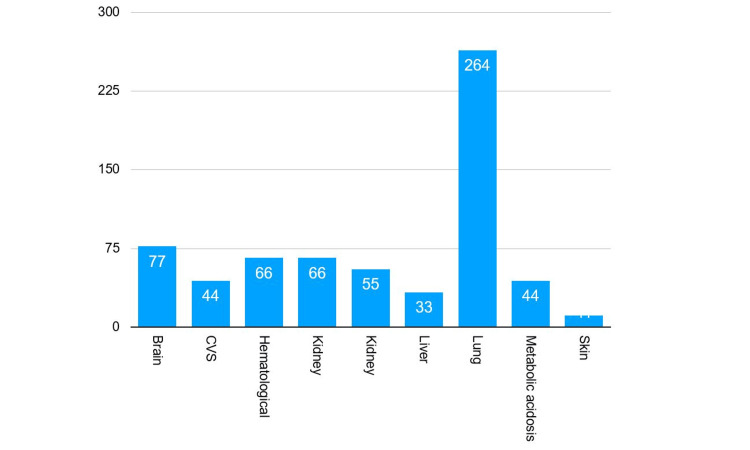
Primary site of infection CVS: cardiovascular system

Figure 2 represents the primary site of infection identified in the majority of patients, with the lungs being the most common site. Pulmonary infections accounted for the largest proportion of sepsis cases, followed by infections originating from the central nervous system (brain), hematological system, and kidneys. Other less frequent primary sites of infection included intra-abdominal infections, skin and soft tissue infections, and bloodstream infections without a clear primary focus. Identification of the primary site of infection is essential for guiding targeted antimicrobial therapy and optimizing patient outcomes.

Mortality outcomes

**Figure 3:**
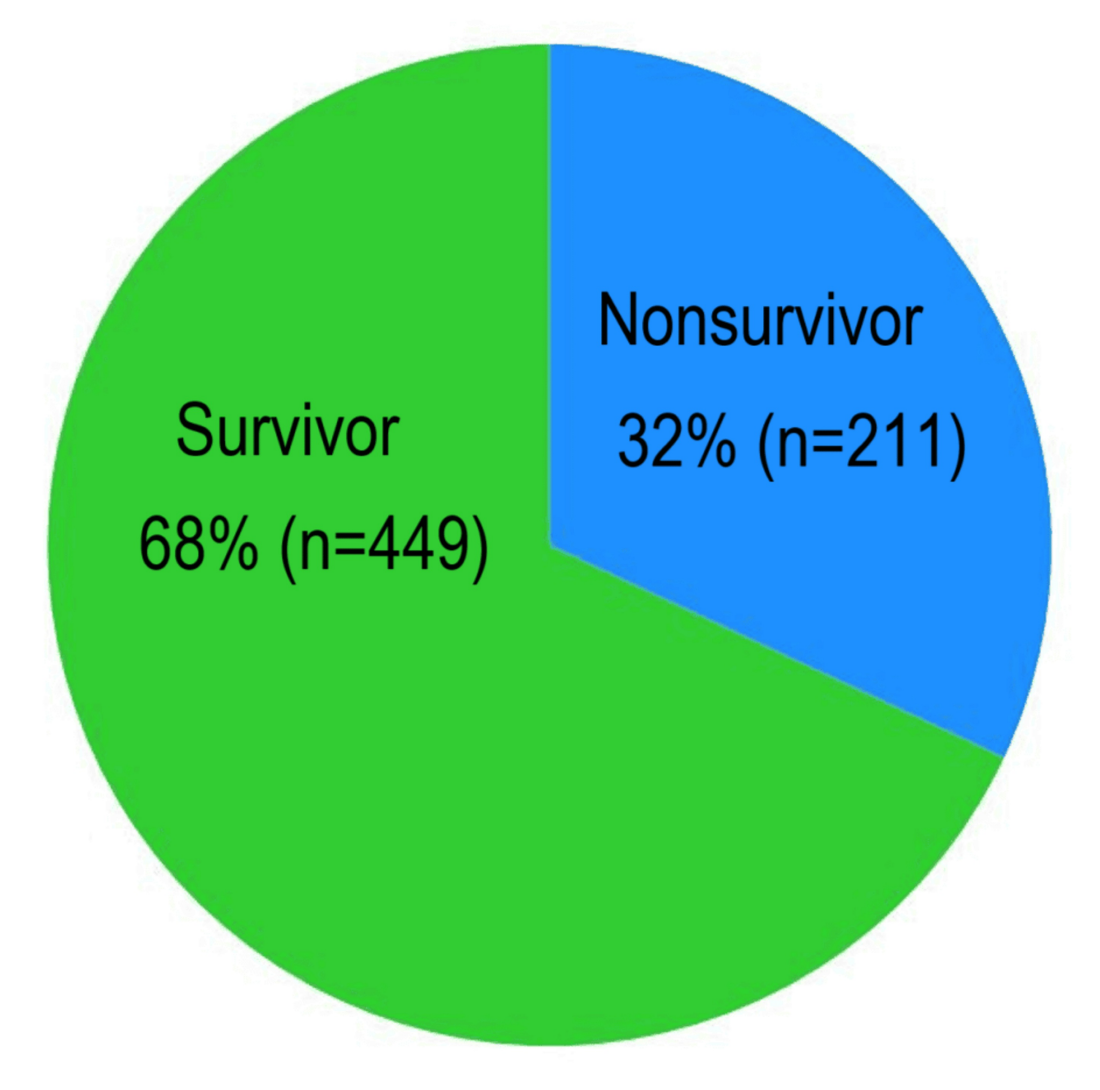
Outcome (N = 660)

Of the 660 patients included in the study, 32% succumbed to their illness, indicating a substantial mortality burden associated with sepsis in the ED setting (Figure 3). This mortality rate is consistent with previously reported global estimates for sepsis-related deaths, particularly in patients presenting with shock or delayed initiation of appropriate treatment. 

The high mortality underscores the critical importance of early recognition and rapid implementation of evidence-based management protocols such as the Sepsis Six bundle. Delayed antibiotic administration, inadequate source control, or incomplete bundle compliance are known contributors to increased mortality, and these factors will be further explored in the discussion section of this study.

## Discussion

In this retrospective cohort study of 660 patients with sepsis presenting to the ED, compliance with the Sepsis Six bundle within the recommended one-hour window was found to be suboptimal, similar to observations from other low- and middle-income countries (LMICs) [3]. The mean age of our cohort (44.8 ± 11.7 years) was younger than reported in Western studies, where the median age often exceeds 60 years [9], likely reflecting the younger demographic structure and higher prevalence of infectious diseases in India. Male sex predominated (63%), a finding consistent with earlier sepsis audits in the region [10].

Nearly half of our patients (43.3%) presented with shock, and two-thirds developed sepsis after 24 hours of admission. This late-onset group is of particular concern as nosocomial infections are associated with higher mortality rates and antimicrobial resistance [11]. The most common primary site of infection was the lung, aligning with findings from the UK Sepsis Trust audit [7]; this differs from several cohort studies, where pulmonary and urinary tract infections predominate [12]. The predominance of pulmonary infections aligns with global epidemiological patterns, where pneumonia remains a leading cause of sepsis, particularly in adult populations [13]. Our study’s mortality rate of 32% is higher than the 20-25% reported in early Sepsis Six implementation studies ([14,15]. This disparity may reflect delayed recognition, resource constraints, and limited critical care capacity.

Multiple studies have demonstrated that each hour’s delay in bundle completion significantly increases mortality risk [16,17]. In our analysis, timely bundle completion was associated with improved survival, echoing previous evidence that rapid implementation of all six interventions, oxygen therapy, blood cultures, intravenous antibiotics, fluid resuscitation, lactate measurement, and urine output monitoring, can improve outcomes [18]. Despite global advocacy for early sepsis care, achieving high compliance remains a challenge. International quality-improvement programmes have shown improved performance; NHS Wales has incorporated Sepsis Six indicators into routine monitoring [19], and "SEPSIS KILLS", a program in New South Wales (Australia), achieved better timeliness in antibiotic delivery [20]. Our findings underscore the need for similar quality improvement initiatives in Indian EDs. Potential strategies include dedicated sepsis response teams, standardized triage alerts, and point-of-care lactate testing.

The limitations of our study include its single-center retrospective design, potential for incomplete records, and lack of long-term follow-up data. However, the large sample size and inclusion of both community- and hospital-acquired cases strengthen the generalizability of our findings.

## Conclusions

The compliance with the Sepsis Six bundle in the ED remains suboptimal, with timely completion of all components within the recommended one-hour window achieved in only a minority of patients. However, higher compliance was associated with improved patient outcomes, including reduced in-hospital mortality and shorter LOS. These findings highlight the critical importance of prompt recognition of sepsis and rapid initiation of evidence-based interventions. Targeted quality improvement measures, staff training, and streamlined ED workflows are warranted to enhance adherence to the Sepsis Six bundle and ultimately improve survival rates in patients with sepsis.
